# Structure, Activity and Function of the NSD3 Protein Lysine Methyltransferase

**DOI:** 10.3390/life11080726

**Published:** 2021-07-21

**Authors:** Philipp Rathert

**Affiliations:** Department of Biochemistry, Institute of Biochemistry and Technical Biochemistry, University of Stuttgart, 70569 Stuttgart, Germany; philipp.rathert@ibtb.uni-stuttgart.de; Tel.: +49-711-685-64388

**Keywords:** NSD3, WHSC1L1, structure and function

## Abstract

NSD3 is one of six H3K36-specific lysine methyltransferases in metazoans, and the methylation of H3K36 is associated with active transcription. NSD3 is a member of the nuclear receptor-binding SET domain (NSD) family of histone methyltransferases together with NSD1 and NSD2, which generate mono- and dimethylated lysine on histone H3. NSD3 is mutated and hyperactive in some human cancers, but the biochemical mechanisms underlying such dysregulation are barely understood. In this review, the current knowledge of NSD3 is systematically reviewed. Finally, the molecular and functional characteristics of NSD3 in different tumor types according to the current research are summarized.

## 1. Introduction

In eukaryotes, DNA is assembled into a higher order nucleoprotein structure called chromatin. Besides the condensation of the DNA, chromatin poses a variety of different functions centered around the regulation of transcription, replication, DNA repair and recombination. The main unit of chromatin is the nucleosome consisting of 147 base pairs (bp) of DNA, which is wrapped around the histone octamer comprising two molecules of each core histone: H2A, H2B, H3 and H4 [1]. The linker histone protein H1 is involved in packaging nucleosomes and proteins such as condensin, cohesin, CCCTC-binding factor (CTCF) or Yin Yang 1 (YY1) to organize the chromatin into higher order structures such as gene loops, topologically associated domains (TADs), chromosome territories, and chromosomes [2,3,4]. Chromatin adopts a highly condensed structure, called heterochromatin, where genes are less accessible and generally transcriptionally silent. In turn, decondensed chromatin, called euchromatin, is much more accessible and harbors the majority of actively transcribed genes [5].

In order to establish or maintain a cell-type-specific gene expression program, the chromatin structures need to be highly dynamic to allow access of transcription factors and other regulatory entities to the DNA at defined time points. These events are tightly regulated by post-translational modifications (PTMs) which are enriched at the unstructured and flexible N-terminal regions of the histone proteins. These histone tails protrude from the nucleosome core and are subject to a diverse array of PTMs, e.g., acetylation, phosphorylation, ubiquitination and methylation, often referred to as the “histone code” that extends the information potential of the genetic code [6,7,8]. The “histone code” hypothesis suggests that specific patterns of modifications function as a barcode and recruit distinct combinations of proteins or protein complexes to drive specific transcriptional programs [9,10].

Histone lysine methylation is among the best characterized PTM of the histone code and is attached to the basic side chains of lysine by a diverse set of sequence-specific lysine methyltransferases [11]. Histone lysine methylation mediates either an activating or repressive effect on gene transcription, which depends on the site, degree of methylation, genomic location, and the status of other coexisting PTMs [11]. The methylation of H3K36 is generally linked to the transcriptionally active state and introduced by six different methyltransferases, which can establish H3K36 methylation to various degrees [12]. The nuclear receptor-binding SET domain (NSD) family of histone methyltransferases is composed of three members of this family, namely NSD1, NSD2/MMSET/WHSC1, and NSD3/ WHSC1L1 (referred to as NSD2 and NSD3 from here on) [13], which all generate mono and dimethylation of lysine 36 on histone H3 (H3K36me1/me2).

NSD3 was first characterized in 2001 as the third member of the NSD gene family [14,15]. Despite the physiologic importance of NSD family proteins, their mechanisms of action are only beginning to become elucidated. In the following review, the structural and functional features of NSD3 will be discussed in more detail with references to the other family members in case information is available.

## 2. Structural Features

The full-length (FL) members of the NSD family of histone methyltransferases are large multidomain proteins, which share most of the evolutionary conserved domains. They belong to the so-called SET domain-containing lysine-specific methyltransferases [16] and the domain involved in the catalytic activity is the SET domain, named after the Su(var)3-9, Enhancer-of-zeste and Trithorax (SET) proteins identified in Drosophila [17]. The SET domain is flanked by the associated with SET (AWS) and post-SET domains.

**Figure 1:**
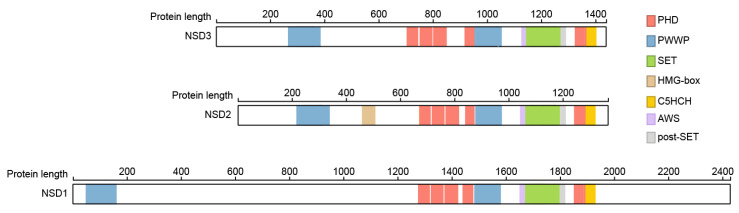
Structural relationship within the NSD family. The major domains of all three members of the NSD family of histone methyltransferases are highlighted. Numbers represent the number of amino acids in each full-length NSD protein. Proteins were extracted using ProteinPaint [33].

Besides the SET domain FL-NSD family members contain two PWWP domains named after its central core Pro-Trp-Trp-Pro motif, a five plant homeo domains (PHD) and a Cys-His-rich domain (C5HCH) domain (Figure 1). Crystal structures showed that the fifth PHD domain (PHD5) and the adjacent Cys-His-rich domain (C5HCH), located at the C terminus of NSD3, fold into a novel PHD-PHD-like module recognizing the unmodified H3K4 and trimethylated H3K9 by PHD5. This function is not conserved between members of the NSD family, with PHD5 of NSD2 showing stronger preference for unmethylated H3K9 (H3K9me0) than trimethylated H3K9 (H3K9me3), and the NSD1 PHD5-C5HCH showed no binding to histone peptides at all [18], but is in involved in binding to the transcription cofactor Nizp1 in NSD1 [19,20,21].

Not much information is available about the specific roles of the other domains of NSD3, and most functions can only be roughly implied from information published for NSD1 and 2. The first N-terminal PWWP domains of NSD1 and 2 were shown to bind to methylated H3K36 to stabilize NSD2, and probably NSD1, at chromatin, and the catalytic SET domain of NSD2 propagates this gene-activating mark to adjacent nucleosomes [22,23,24,25,26,27].

The PHD1-3 motifs of NSD2 were shown to be important for its H3K36me2 methylation activity. Specifically, the removal of PHD1 decreased H3K36me2 activity and PHD2 caused NSD2 localization into the cytoplasm, which resulted in a complete loss of activity [28]. More details are known for the PHD domains of NSD1. These were shown to mediate binding of NSD1 to methylated H3K4 and K9 with a preference for dimethylated lysines in vitro [21]. Only the PHD4, PHD5 and C4HCH domains show binding to both modifications, which is controversial as both methylation states are associated with opposite transcriptional states [29,30,31]. The binding of various states of H3K4 and H3K9 methylation would allow NSD1 to recognize genes in stages of transcriptional activation and repression. It was therefore hypothesized that the activities of NSD1 cofactors would ultimately lead to either the enforcement, or alternatively, to the reversal of repression mechanisms [21]. 

All three members of the NSD family of histone H3K36 methyltransferases share most of the common motifs except NSD2, which contains a so-called high mobility group (HMG) domain. The HMG domain of NSD2 was shown to interact with the DNA-binding domain of the androgen receptor (AR), thereby enhancing the nuclear translocation of both proteins [32]. Future studies are necessary to reveal whether the common corresponding domains of NSD3 have similar roles.

## 3. NSD3 Structure

The structure of the full-length NSD3 protein was never solved completely until now, due to its large protein size. An NSD3 construct containing amino acids 1054–1285, which spans the entire catalytic SET domain and additional residues on both sides without the reader domains, was crystallized in the presence of a histone H4 sequence flanking lysine 44 (H4K44), in which K44 was replaced by the unnatural amino acid norleucine (Nle) [34]. The catalytic part of NSD3 folds into a compact globular structure [34], which was confirmed later using cryo-electron microscopy (cryo-EM) studies on a larger version of NSD3 containing the C-terminal part of NSD3 starting from the first PHD domain (termed NSD3C) in complex with the nucleosome [35]. The histone peptide binds in a narrow groove and the lysine is occupying the substrate lysine channel. Interactions between the H3 tail and the SET domain are mainly mediated by hydrogen bonds, which tightly position the target lysine of H3 within the catalytic pocket. The hydrophobic side chain of the lysine points towards the methyl donor S-adenosylmethionine (SAM) through insertion into a hydrophobic pocket. [30]. The structures currently available for the NSD family show that a loop connecting the SET and post-SET domains can adopt multiple conformations, which are important for the regulation of the catalytic activity. This loop can extend over the H3 tail binding site of the SET domain, leading to autoinhibition [34,35] and significant reorganization of the autoinhibitory loop is observed in the structure of NSD3. In complex with the peptide, the autoinhibitory loop moves towards the C-terminus, which opens the substrate binding site for the peptide [34]. Similar to NSD3, the NSD1 and two post-SET domains are attached to the catalytic SET domain via an autoinhibitory loop region and inhibition is relieved upon nucleosome binding [13,36].

**Figure 2:**
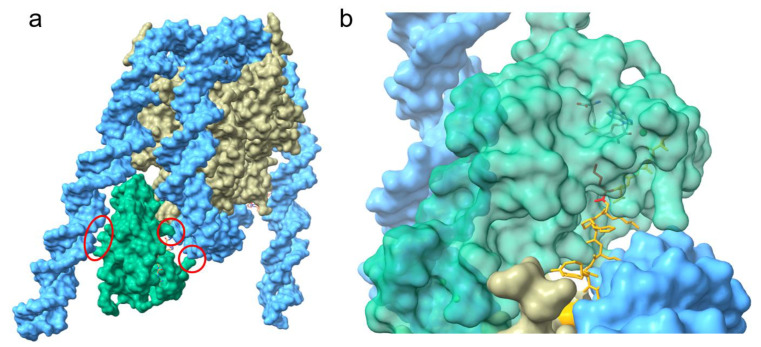
Surface representation of the structure of NSD3 bound to the nucleosome core particle (adopted from 7CRR PDB [35]). (**a**), NSD3 forms several contacts with the nucleosomal DNA (highlighted with red circles) and inserts between the DNA and the octamer. (**b**), Magnified view of the catalytic center of NSD3 showing the norleucine at position 36 is oriented in the catalytic center of NSD3. NSD3 is colored in green, the octamer in grey and the nucleosomal DNA in cyan. The bound H3 tail is shown in orange and the norleucine (inserted in the catalytic pocket of the SET domain) is depicted in red.

The recent cryo-EM studies provided a more detailed view on the importance of the nucleosome-bound DNA in the activation of NSD3 [35]. NSD3 forms several contacts with the nucleosomal DNA and inserts between the histone octamer and the DNA near the linker region leading to an unwrapped segment of DNA [35] (Figure 2a). The interactions between NSD3 and the unwrapped DNA are required for the full activity of NSD3 and several basic residues from the long N-terminal loop bind to the unwrapped segment of DNA. This interaction of NSD3 to the DNA is strengthened by additional salt bridges between lysine and arginine residues of the SET and post-Set domain and the phosphate backbone [35]. Interactions within this region of DNA not only stabilize the binding between NSD3 and the nucleosome core particle (NCP), but also enable the positioning of the H3 tail in the substrate-binding groove of the SET domain (Figure 2b). The interaction of NSD3 with the DNA at several positions, which leads to the partial unwrapping of the DNA, is essential for the correct positioning of K36 in the active center and is a key factor that determines NSD3 substrate specificity. Additionally, NSD3 makes extensive intermolecular contacts with a short section of the C terminus of histone H2A as well as a long fragment of H3 that contains the first α-helix and the N-terminal tail. 

Furthermore, the AWS domain extends into the core histones and contacts the H2A C-terminal fragment through hydrophobic and electrostatic interactions. These contacts result in an extended conformation of NSD3, rendering NSD3 catalytically active and contributing to the precise positioning of NSD3 to specifically bind H3K36. However, it is possible that the conformational states observed differ with the full-length protein when compared to truncated constructs, which could influence the regulation of the enzyme activity by the autoinhibitory loop. 

Additionally, the C-terminal part of NSD3 the crystal structure of the PWWP1 domain of NSD3 (residues 247–398) was solved and revealed a classical PWWP domain fold, as described previously [37,38]. An N-terminal β-barrel of 5 antiparallel β-strands (β1–β5), with a short helix insertion between β4 and β5 is followed by 3 α helices. The aromatic cage is formed by the aromatic amino acids Trp284, Tyr281, and Phe312, which are located at flexible loops connecting the different β-sheets. The aromatic cage could potentially accommodate an H3 peptide methylated at K36, indicated by the superimposition of the BRPF1-PWWP domain in complex with an H3K36me3 peptide [37,39].

## 4. Biochemical Features

The catalytic activity of the NSD family of histone H3K36 methyltransferases is restricted to a lower degree methylation of H3K36, and a specificity for mono and dimethylation is observed [12,40]. The substrate specificity of the NSD family of histone methyltransferases has long been debated and in vitro the catalytic domain (CTD) of NSD1, NSD2, and NSD3 were shown to recognize and methylate H3K4, H3K9, H3K27, H3K36, H3K79, and H4K20 peptides, with substantial differences in catalytic activities depending on the substrate [25]. NSD3 had previously been reported to specifically methylate H3K4 and H3K27 [41]. However, additional data with recombinant nucleosomes as substrate showed that the SET domains of all NSD family members specifically methylated K36 on histone H3. In contrast, when using recombinant histone octamers as substrate, the activity of NSD3 remained specific for H3 although with much lower activity, whereas the NSD2-SET domain mainly targeted H4 with very weak activity on H3 and the NSD1-SET domain methylated all components of the octamer, namely histone H3, H2A/H2B, and H4. Therefore, it was proposed that DNA acts as an allosteric effector of the NSD family proteins, such that H3K36 becomes the preferred target [42], which was recently confirmed through structural analysis [35].

Apart from the regulation of their enzymatic activity through binding of the nucleosome and the resulting clearance of the catalytic site from the autoregulatory loop, all members of the NSD family of histone methyltransferases are inhibited in their activity by different post translational modifications (PTMs) on histones. The ubiquitination of histone H2A at Lys119 [35,43] inhibits the activity of the whole NSD family of methyltransferases, which could be explained by the fact that they form extensive intermolecular contacts with the C terminus of histone H2A described for NSD3 [35]. Furthermore, the trimethylation of H3 at Lys4 also decreased the catalytic activity of NSD3, which correlates with the finding that the last PHD finger of NSD3 favors an unmodified Lys4 of H3 [18]. This suggests that binding of the unmodified H3 tail at lysine 4 contributes to some extent to the catalytic activity of NSD3. By contrast, the trimethylation of H3 at Lys27 did not alter the catalytic activity of NSD3 [35], which is intriguing because K27me3 rarely co-exists with K36me2 or K36me3 on the same histone. H3 polypeptide and PRC2 activity is greatly inhibited on nucleosomal substrates with preinstalled H3K36 methylation [44,45].

## 5. Cellular Features

**Figure 3:**
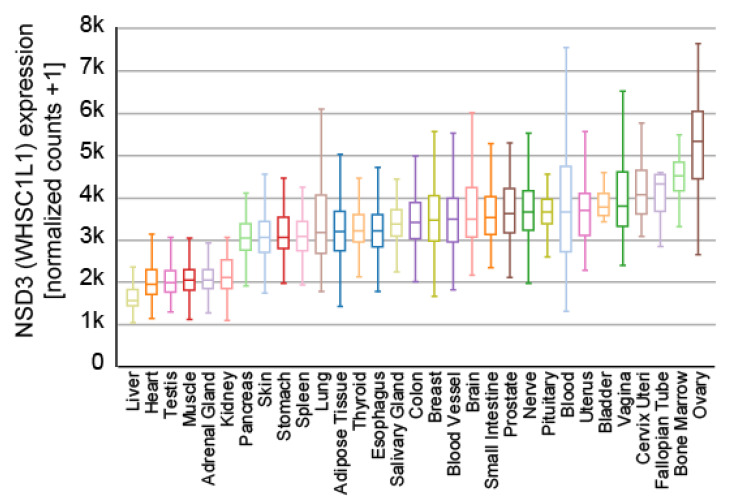
Expression of NSD3 across different human tissues. The data used for the analyses were obtained from the GTEx Portal (9783 samples). Visualization was generated with the UCSC Xena platform [46].

**Figure 4:**
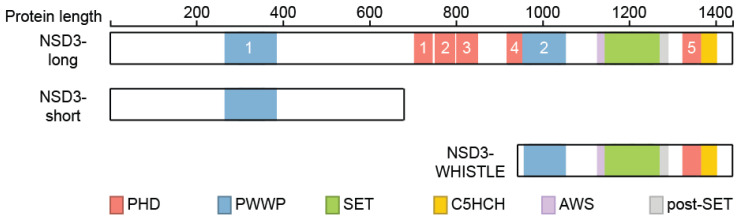
Isoforms of NSD3. Three isoforms of NSD3 are depicted and their domains were highlighted. Numbers of the different domains are indicated.

NSD3 is ubiquitously expressed (Figure 3) and generates three major transcripts, a long (NSD3-long) isoform of 1437 amino acids, a short (NSD3-short) isoform containing 645 amino acids [14,15] and another short transcript called WHSC1-like 1 isoform 9 with methyltransferase activity to lysine (WHISTLE), which consists of 506 amino acids (Figure 4) [41].

The NSD3-short protein lacks the catalytic SET domain and only contains the amino-terminal PWWP domain (Figure 2) [15] that binds to histone H3 when it is methylated on lysine 36 before [22]. NSD3-short was shown to interact with the bromodomain-containing protein 4 (BRD4) [47,48,49], which belongs to the bromodomain and extra-terminal domain (BET) protein family [50]. BRD4 plays an important role in controlling oncogene expression and genome stability and has sparked considerable interest as a drug target in multiple diseases in the past few years [51,52,53]. NSD3-short interacts with the extra terminal (ET) domain of BRD4 [48,49], which functions as an adaptor protein that links BRD4 to the chromatin remodeler CHD8 to enable transcriptional programs [48].

Both the NSD3-long and NSD3-short transcripts are co-expressed in many tissues [14,15], whereas WHISTLE was found to be mainly expressed in testis and in bone marrow mononuclear cells of AML and ALL patients [41]. In contrast to NSD3-long, WHISTLE only contains the second PWWP, SET, and post-SET domains (Figure 4) and was reported to facilitate transcriptional repression through its enzymatic activity and by recruiting HDACs [54], which is controversial to some extent, as all other reports connect NSD3 to transcriptional activation.

All NSD family proteins show methylation activity towards H3K36, which is restricted to mono and dimethylation [12,40]. Numerous studies in multiple systems support a role for H3K36 methylation in transcriptional activation [55,56]. While H3K36me3 exhibited characteristic enrichment within gene bodies, H3K36me2 shows a very distinctive genomic occupancy pattern and displays a significant enrichment in promoters and intergenic regions in various cell types [24,57,58] suggesting that H3K36me2 might play a role in enhancer regulation. Evidence for the function of H3K36me2 in the regulation of enhancer accessibility was provided recently through the investigation of Nsd1-mediated H3K36me2 distribution [45,58]. Interestingly, the simultaneous presence of H3K36me2 and H3K27me2, which is regulated through the activity of the polycomb repressive complex 2 (PRC2) [59], strongly correlate in embryonic stem cells (ESCs), whereas H3K36me3 and H3K27me3 are anticorrelated [45]. A switch from di- to trimethylation at K36 induces an increase in H3K27me3 [45], which results in the downregulation of the enhancer activity. In line with this observation, NSD2 was shown to regulate epithelial plasticity by altering enhancer activity. H3K27ac peaks residing within intergenic H3K36me2 domains are lost when H3K36me2 levels decrease, providing another indication that H3K36me2 mediates its effects by modulating enhancer activity [60]. Due to its comparable substrate specificity and structural similarity, an analogous function could be conceived for NSD3 as well, but this needs to be investigated experimentally.

Furthermore, H3K36me2 is required for recruitment of DNMT3A and maintenance of DNA methylation at intergenic regions [58]. Genome-wide analysis showed that the binding and activity of DNMT3A co-localize with H3K36me2 at non-coding regions of euchromatin [58]. Accordingly, the PWWP domain of DNMT3A shows dual recognition of H3K36me2/3 in vitro with a higher binding affinity towards H3K36me2 [58,61]. However, ChIP-seq experiments investigating different lysine methylation states should be taken with great care. Many antibodies which are raised against a specific methylation state can show high cross-reactivity to other states at the same lysine residue [62]. Until now, it was unclear whether NSD3 contributes to the above-mentioned deposition of H3K36me2 at intergenic regions in other cell types where its expression is dominant over NSD1 and NSD2 or if the activity of NSD3 is restricted to other regulatory genomic elements.

Analogous to other known lysine methyltransferases [63], members of the NSD family were shown to methylate non-histone proteins. Apart from histone substrates, NSD3 recently was reported to methylate the epidermal growth factor receptor (EGFR), leading to enhanced activation [64], and NSD1 was shown to mono- and dimethylate p65, an NF-κB family transcription factor, at K218 and K221, which stimulates the expression of p65-dependent tumorigenic genes [65]. Furthermore, NSD1 was shown to methylate histone H1 in a variant-specific manner [66].

## 6. The Role of NSD3 in Cancer

Knowledge about the function of NSD3 in individual diseases is sparse, and most of the information available is about its role in different tumors. NSD3 is located on chromosome 8p11.2, in a region which has been linked to various diseases and that is amplified in primary tumors and cell lines from breast carcinoma [14,15]. As well as NSD3, the 8p11.2 region contains a set of genes including TAM, FGFR1, and LETM2 [15,67].

Genomic alterations of NSD3 occur in multiple cancer types, implicating its cancer-promoting role [12,68]. In most cases, the fusion between the NUP98 and NSD3 genes was detected in patients with AML or myelodysplastic syndrome [69,70], which promotes hematopoietic transformation in the same fashion as already shown for the NUP98-NSD1 fusion protein, due to the structural similarity between the two [71]. Besides the fusion to NUP98, NSD3 fusion has been observed with NUTM1 in primary pulmonary NUT carcinoma [72,73,74], which is known to typically harbor the BRD4/3-NUT fusion oncoprotein [75].

In line with the function of NSD3-short as an adaptor protein of BRD4 and CHD8 [48], MLL-AF9 rearranged acute myeloid leukemia (AML) were proven to be dependent on NSD3 [48,51,76]. This was confirmed by the development of a chemical probe for the PWWP1 domain of NSD3, which leads to the reduced proliferation of AML cell lines through the downregulation of MYC mRNA [37].

**Figure 5:**
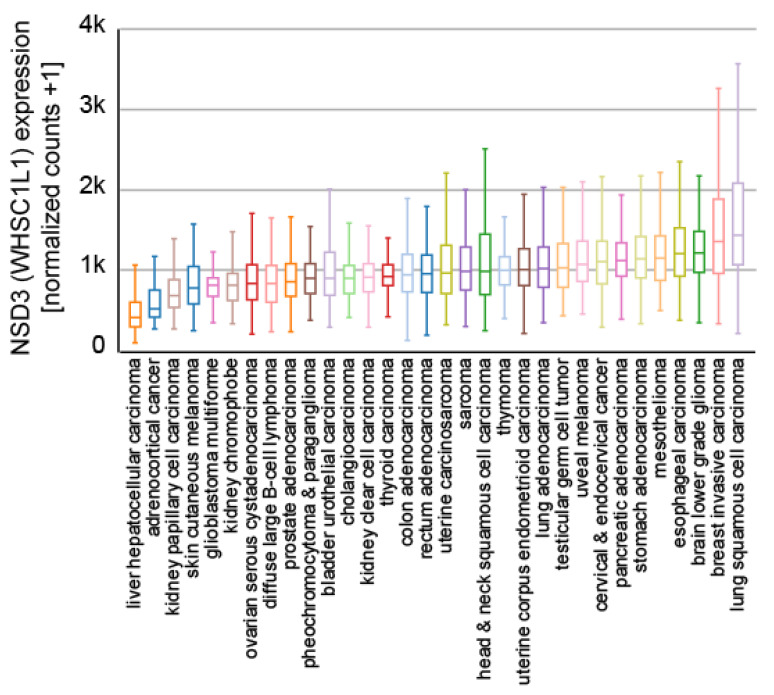
Expression of NSD3 in different indicated cancer subtypes. Samples from 9621 primary tumors of the TCGA Pan-Cancer set (https://www.cancer.gov/tcga, accessed on 21 July 2021) are presented and visualized with the UCSC Xena platform [46].

In addition, the 8p11.2 region is amplified in many cancers [67], leading to the increased expression of NSD3 (Figure 5), and reports have described NSD3 to be essential for tumor maintenance and the suppression of NSD3 expression leads to reduced cell proliferation in lung cancer [77,78,79], breast cancer [80,81], and osteosarcoma [82]. Furthermore, the 8p11.2 region is amplified in breast cancer (BC) [14,80,81] and the overexpression of NSD3 is linked to overexpression of the estrogen receptor alpha (ERα) in breast cancer [80]. A similar scenario was described for colorectal cancer (CRC) [83]. Here, NSD3 was shown to be upregulated in CRC and the suppression of NSD3 expression resulted in a decrease in proliferation, migration, and EMT marker proteins such as E-cadherin and N-cadherin [83].

Thus far, only one non histone protein has been described, which is methylated by NSD3 [64]. The epidermal growth factor receptor (EGFR) was shown to be methylated by NSD3, leading to the enhanced activation of the associated ERK cascade without stimulation by EGF. In addition, nuclear EGFR was showed to enhance its interaction with proliferating-cell-nuclear-antigen (PCNA) resulted in enhanced proliferation in squamous cell carcinoma of the head and neck (SCCHN) [64].

**Figure 6:**
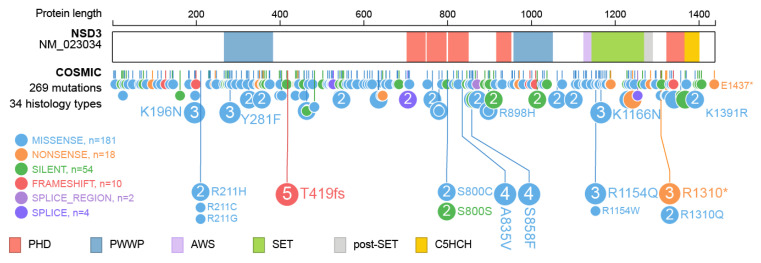
Mutations observed within NSD3. Visualization of NSD3 mutations listed in the Catalogue of Somatic Mutations In Cancer (COSMIC release 87) using ProteinPaint [33]. The major domains NSD3 are highlighted. Mutations are color coded as indicated. Numbers represent the number of amino acids.

Furthermore, over 260 mutations have been described within the NSD3 protein (Figure 6) and for most, the underlying change in protein function has not yet been described. Intermolecular contacts between NSD3 and nucleosomes are altered by several recurrent cancer-associated mutations. E1181K and T1232A substitution leads to enhanced enzymatic activity through preventing the autoinhibitory loop from blocking the active site, which improves the insertion of the target H3K36 into the catalytic pocket of NSD3 [35,79]. Both mutations were demonstrated to promote the proliferation of cancer cells and accelerated growth of xenograft tumors [35]. There is no specific information available on the effect of the other mutations observed in NSD3.

Besides mutations in NSD3 itself, so-called onco-histones harboring mutations of the lysine at position 36 [84,85,86,87], lead to alterations of the function of NSD3. Given the importance of H3K36me2 in maintaining active enhancers to regulate epithelial-to-mesenchymal identity, tumor differentiation, and metastasis [45,60] it is inevitable that these onco-histones impose a strong negative impact on transcriptional maintenance. The incorporation of a lysine-to-methionine histone H3 mutant (H3K36M) led to a genome-wide reduction in H3K36me2 and H3K36me3 levels in different malignancies [60,86,87,88,89], which was attributed to a direct inhibitory effect of the H3.3K36M mutation on NSD2 and SET Domain Containing 2 (SETD2) [87]. Unfortunately, in these studies, the effect of the K36M mutation was not tested on NSD3 activity, but the comparable substrate specificities and structural similarities suggest a potential inhibitory effect on NSD3 as well.

Two recent publications shed more light on how altered NSD3 activity promotes tumor development and growth. These studies investigated the role of NSD3 in squamous cell lung cancer [79] and breast cancer [80]. Both showed that NSD3 acts as a factor that reprograms the chromatin landscape to promote oncogenic gene expression signatures. Elevated NSD3 expression [80] or hyperactivity [79] leads to an increase in H3K36me2 which inhibits the activity of the PRC2 complex [45]. This leads to the reexpression of developmental genes like MYC [79] or Notch3 [80], which promote stem cell like properties and in turn malignant transformation [79,80]. 

## 7. Outlook

Despite the recent achievements in the structural and biochemical analyses of NSD3 in complex with the nucleosome, which provided a molecular basis for the nucleosomal preference and activation mechanism of NSD proteins, not much information is available on cellular functions of NSD3 itself. Nevertheless, the fact that the methylation of H3K36 plays such an important role in regulating enhancer activity [45,60] and NSD3 is amplified in many cancers [14,67,73,77,79,80], suggests that NSD3 must play an important role in many different cellular processes. Epigenetic-based therapies are emerging as effective and valuable approaches in cancer and targeting NSD3 may indeed present a valuable approach [37,48,51,76]. However, the existence of at least six histone methyltransferases, which are capable of methylating H3K36, complicate the efforts in understanding the effects of NSD3 in cells, and further work will be needed to clarify these roles.

## Data Availability

Not applicable.
